# BPSM-D-19-00022R2 Subjective well-being and problem-solving skills for alleviating the stress of elderly men attending a randomized controlled trial of shogi-assisted cognitive behavioral therapy

**DOI:** 10.1186/s13030-019-0153-4

**Published:** 2019-05-09

**Authors:** Mutsuhiro Nakao, Hirokazu Furukawa, Chiho Kitashima, Shota Noda

**Affiliations:** 1 Department of Psychosomatic Medicine, School of Medicine, International University of Health and Welfare, 4-3, Kozunomo, Narita-shi, Japan; 2 School of Basic Research and Improvement of Practice for Education, Naruto University of Education, Tokushima, Japan; 3 Graduate School of System Design and Management, Keio University, Tokyo, Japan; 4 Graduate School of Human and Social Sciences, Musashino University, Tokyo, Japan

**Keywords:** Board game, Happiness, Shogi, Stress, Well-being

## Abstract

Shogi is a popular board game in Japan, and shogi-assisted cognitive–behavioral therapy (S-CBT) has been applied in Kakogawa City, a Japanese municipality. The purpose of this study was to clarify the effects of S-CBT on the subjective well-being of elderly men. Participants were 61 elderly men with amateur skill at shogi. They were randomly assigned to either the S-CBT group or a wait list group (control). The S-CBT group participated in a weekly, six-session S-CBT program. The intervention outcomes were scores on the K6, Lubben Social Network Scale, and a five-item cognitive–behavioral functioning scale. The Subjective Well-being Scale was used to assess happiness and satisfaction with life, and all the participants were classified into high- and low-happiness groups using the median score as the cutoff. The results showed that scores on “self-reinforcement” were significantly (*P* < 0.05) increased for those receiving S-CBT compared with controls, regardless of the participants’ happiness scores. In contrast, the scores on “problem solving skills for alleviating stress” were significantly (P < 0.05) increased for those receiving S-CBT compared with controls only among those in the low-happiness group. These results remained significant after controlling for the effects of age and baseline scores on the K6, Social Network Scale, and “problem solving skills for alleviating stress” category. The S-CBT may be especially beneficial when focused on practical advice for the stress management of older people with low subjective well-being. (trial registration: 000036003 [UMIN, Japan]).

**Trial Registration:** Trial registration number: 000036003 [UMIN, Japan].

## Introduction

Shogi is a traditional Japanese board game that is played on a 9 × 9-square board, unlike the 8 × 8-square board used in chess [1]. Some shogi pieces are very similar to chess pieces, but the game differs from chess mainly in allowing players to keep captured pieces and replay them as their own. Players must therefore make quick and strategic decisions in choosing to attack or defend at any given board position. These decision-making processes may be beneficial for improving logical thinking and non-verbal communication during play, which may, in turn, enhance reasoning about their own and their opponents’ perspectives in their efforts to win the game [2]. Furthermore, as a leisure-time activity, shogi may also serve as a form of stress management, as the fight-or-flight response is regulated safely within the sophisticated structures of match-type games [2]. With these considerations as background, neurological changes in brain functioning during shogi play have been intensively examined during the past decade [3, 4].

Cognitive–behavioral therapy (CBT) helps individuals to recognize and modify irrational beliefs, such as, “There is nothing I can do about the problem I am facing.” [5] Compared with many other countries, CBT is not yet common in Japan. Thus, a shogi-assisted CBT (S-CBT) program was developed [6] to make CBT more interesting and, thereby, enhance its acceptance, especially among those who are unwilling to consult a psychologist and undertake CBT. When S-CBT is provided in the context of an enjoyable activity that connects CBT to actual situations that arise in playing shogi, participants may experience less resistance to the psychological intervention and may increase their positive thoughts [7–10].

To clarify the impact of S-CBT on cognitive–behavioral functioning, mental health, and social support skills, a randomized controlled trial was conducted with volunteers who enjoyed playing shogi. In the present study, we were interested in psychosocial variables related to happiness or, more broadly, subjective well-being (SWB), as low or absent SWB is a risk factor for future physical and psychiatric disorders [11]. Research on the correlates of SWB across the life course has shown that social relationships figure more prominently for older than for younger individuals [11]. Thus, we examined the effect of S-CBT in groups of participants with high and low SWB to develop ideas for promoting social life skills among elderly people, especially those with low SWB.

## Methods

### Participants

Participants were recruited from among residents living in Kakogawa City, Hyogo, Japan, via public announcements. The inclusion criteria admitted adults over 60 years of age who were skillful at shogi, who had attended the information session for this trial, and who provided written informed consent. Exclusion criteria included medical morbidity such as dementia that would interfere with completion of the battery of self-rated questionnaires; no one was excluded from the present study. Patients were assigned to the intervention or control arm following a baseline assessment at Kakogawa Shogi Plaza. Those allocated to the control group were instructed to return to the same place after 6 weeks to complete a follow-up assessment, after which they received the same S-CBT program. The study was approved by the Human Subjects Committee at the university and was conducted between September and December 2017.

### Measures

A battery of four questionnaires was administered to participants just before and again after the 6-week intervention period. The battery included the following measures.

The K6 was used to measure psychological distress [12]. The K6 is a 6-item inventory drawn from depression and anxiety related symptomology. Participants responded using a five-point Likert-type scale (0 = never to 4 = always). The K6 is a truncated version of the K-10; its purpose is to function as a global measure of distress. The total scores, which ranged from 0 to 20, were analyzed.

The Japanese version of the abbreviated form of the Lubben Social Network Scale was used as a measure of social support [13]. The scale is a 3-item inventory for measuring the number of one’s friends. Participants responded using a six-point Likert-type scale (0 = none to 5 = more than nine) and were then grouped into three patterns of social support. The total scores ranged between 0 and 15.

To assess cognitive–behavioral patterns, five items were used, each corresponding to one session of the S-CBT program. These included distraction activity, behavior inhibition, problem solving skill, self-reinforcement, and negative automatic thoughts [14]. The tendencies represented by each cognitive pattern were rated on a five-point scale (1 = never to 5 = often).

The Subjective Well-being Scale (SWB scale) was used to assess feelings of happiness and satisfaction with life [15, 16]. A draft of the SWB scale included 39 positive scales collected through a literature search. Two scales, the “Interdependent Happiness Scale” and the “Satisfaction with Life Scale”, met the criteria for addressing the concerns of this study, and a six-item scale using a five point-scale (1 = very unlikely to 5 = very likely) was developed with a consideration of Japanese cultural characteristics. Thus, the total scores ranged between 5 and 30.

### Procedure

As described in greater detail in a previous study [14], the S-CBT program was carried out in a group format over six weekly, 90-min sessions. In brief, after the first 45 min, which featured a female shogi player’s commentary on the game, the cognitive behavioral intervention was carried out for the last 45 min. Each group was facilitated by a licensed clinical psychologist with 15 years’ experience in CBT. The six S-CBT sessions addressed the following topics: “What kind of conversation skills are necessary for enjoying shogi with many others?” (social support); “How to interrupt your negative ideas when you are losing at shogi and beginning to feel deflated” (distraction activity); “How to act positively when you are losing to an opponent in shogi and becoming deflated” (behavior activation); “How to create a solution when you do not know what shogi move to make next” (problem-solving skills); “The trick of continuing to try to challenge a strong opponent in shogi even when it feels stressful” (self-reinforcement); and “The trick of looking for a solution while considering what has been lost” (negative automatic thoughts).

### Data analysis

All tests of statistical significance were interpreted using a criterion of *P* < 0.05, two-tailed. The participants were classified into high- and low-SWB groups, using the median score on the SWB scale as the cutoff. For continuous variables at baseline, t-tests were used to compare the high- and low-SWB groups. Three-way analyses of variance (ANOVA) were used to evaluate intervention outcomes, using SWB (high or low group), intervention (intervention or control group), and time (pre- or post-intervention) as independent variables. The scores obtained from the CBT and control groups on the outcome measures were compared at the 6-week follow-up assessment. Planned contrasts comparing scores at baseline with those obtained at the 6- week follow-up assessment were calculated for each score; group differences in the change scores were estimated, adjusting for scores at baseline [17]. This approach applies to analysis of covariance (ANCOVA), which, despite its name, is a regression method. The details are as follows: “two parallel straight lines are obtained relating the outcome score to the baseline score in each group, and can be summarized as a single regression equation: follow-up score = constant + a × baseline score + b × group, where a and b are the estimated coefficients and group is a binary variable that is coded 1 for the treatment and 0 for the control. Coefficient b is the effect of interest—the estimated difference between the two treatment groups [17]”. Also, standardized effect sizes (Cohen’s d) [18] were calculated to allow comparisons of effect sizes across different measures and studies. Effect sizes of approximately 0.2, 0.5, and 0.8 are generally considered to represent small, medium, and large effects, respectively [18, 19]. Differences in the change scores and standardized effect sizes between the CBT and control groups were estimated for the high- and low-SWB groups. All analyses were performed using the SAS statistical package [20].

## Results

### Characteristics of participants

A total of 61 subjects who completed the SWB scale with no missing data were analyzed in this study; The characteristics of the sample are summarized in Table 1. The average score (SD) on the SWB scale was 20.2 (4.4), and the median was 21, separating the low- (< 21) and high- (> 21) groups. The K6 scores were significantly greater in the low-SWB group than in the high-SWB group. Scores on the cognitive–behavioral and social support scales were not significantly different between the two groups.

**Table 1:** Group differences in psychosocial and cognitive variables between elderly men with lower scores on the subjective well-being scale (SWB) and those with higher SWB scores

		Group comparison, t-test		
Means (standard deviations)	Total sample (*n* = 61)	Low SWB group (*n* = 30)	High SWB group (*n* = 31)	*P* value^a^
Age, years	72.2 (6.0)	72.9 (5.4)	71.5 (6.6)	0.364
Mental health status, K6 (score range: 0–20)	5.03 (3.75)	6.40 (4.04)	3.71 (2.96)	0.004
Social network scale (score range: 0–15)	7.52 (3.56)	7.83 (3.30)	7.23 (3.87)	0.513
Cognitive behavioral pattern (score range: 1–5)				
Distraction activity when depressed	3.28 (1.27)	3.33 (1.22)	3.23 (1.33)	0.743
Behavior inhibition when depressed	2.48 (1.06)	2.63 (1.19)	2.32 (0.91)	0.255
Problem solving skill against stress	3.23 (1.04)	3.13 (0.90)	3.32 (1.17)	0.482
Self-reinforcement	2.74 (0.96)	2.70 (0.84)	2.77 (1.09)	0.767
Negative automatic thought	3.03 (1.22)	3.17 (1.29)	2.90 (1.16)	0.405

^a^*P* value was based on t-test, two-tailed

### S-CBT effects

The results for the intervention outcome measures are shown in Table 2. The three-way ANOVA (i.e., SWB, intervention, and time) of the total sample found significant effects only for SWB (F value = 20.9, *P* < 0.001) in the model to explain the variance in the K6 scores. None of the three independent variables had significant effects in the model of the Social Network Scale. Concerning cognitive behavioral functioning, significant effects were found only for time (F value = 9.80, *P* < 0.001) in the “distraction activity when depressed” model. Significant effects were found for both SWB (F value = 5.77, *P* = 0.018) and time (F value = 13.2, *P* < 0.001) in the “behavioral inhibition when depressed” model, and for both SWB (F value = 5.82, P = 0.018) and time (F value = 17.7, P < 0.001) in the “problem solving skill against stress” model. Both intervention (F value = 7.06, *P* = 0.009) and time (F value = 8.51, *P* = 0.004) were significant in the “self-reinforcement” model, and both SWB (F value = 4.86, *P* = 0.030) and time (F value = 12.4, P < 0.001) were significant in the “negative automatic thought” model.

**Table 2:** Changes in psychosocial and cognitive variables for Shogi-assisted cognitive behavioral therapy (S-CBT) and control group, according to the levels of subjecvtive well-being (SWB)

	Scores at baseline and follow-up		Change scores from baseline	
	SCBT group	Control group	Adjusted SCBT-Control difference (95%CI)^a^	Effect size
	Mean (SD)	Mean (SD)		(Cohen’s d)^b^
Low SWB group (n = 30)	(*n* = 16)	(*n* = 14)		
Mental health status, K6 (total score range: 0–20)				
Baseline	7.00 (3.79)	5.71 (4.34)	–	–
Post-intervention	6.15 (4.43)	4.09 (3.91)	1.72 (− 1.26, 4.69)	0.18
Social network scale (score range: 0–15)				
Baseline	8.63 (3.83)	6.93 (2.40)	–	–
Post-intervention	7.69 (3.72)	7.00 (3.63)	−0.47 (−2.74, 1.80)	− 0.15
Cognitive behavioral functioning (score range: 1–5)				
Distraction activity when depressed				
Baseline	3.13 (1.26)	3.57 (1.16)	–	–
Post-intervention	3.54 (1.20)	3.00 (1.26)	0.90 (−0.01, 1.81)	0.46
Behavior inhibition when depressed				
Baseline	2.63 (1.36)	2.64 (1.01)	–	–
Post-intervention	2.15 (1.14)	2.64 (0.81)	−0.44 (−1.28, 0.39)	− 0.12
Proble solving skill against stress				
Baseline	3.00 (0.82)	3.29 (0.99)	–	–
Post-intervention	3.85 (0.90)	2.91 (0.94)	*1.01 (0.27, 1.74)*	0.50
Self-reinforcement				
Baseline	2.88 (0.81)	2.50 (0.85)	–	–
Post-intervention	3.77 (1.01)	2.73 (1.01)	*0.92 (0.08, 1.76)*	0.32
Negative autonomic thought				
Baseline	3.50 (1.32)	2.79 (1.19)	–	–
Post-intervention	2.92 (1.44)	3.00 (1.10)	−0.29 (−1.24, 0.66)	− 0.19
High SWB group (n = 31)	(*n* = 17)	(n = 14)		
Mental health status, K6 (total score range: 0–20)				
Baseline	3.76 (3.07)	3.64 (2.92)	–	–
Post-intervention	4.00 (3.44)	4.62 (4.19)	−0.68 (−2.82, 1.45)	− 0.13
Social network scale (score range: 0–15)				
Baseline	7.12 (4.01)	7.35 (5.14)	–	–
Post-intervention	6.93 (2.74)	6.77 (2.77)	0.40 (−1.37, 2.17)	0.15
Cognitive behavioral functioning (score range: 1–5)				
Distraction activity when depressed				
Baseline	3.12 (1.36)	3.36 (1.34)	–	–
Post-intervention	4.07 (0.70)	3.69 (1.11)	0.43 (−0.24, 1.11)	0.23
Behavior inhibition when depressed				
Baseline	2.35 (0.93)	2.29 (0.91)	–	–
Post-intervention	1.73 (0.80)	2.46 (0.78)	−0.73 (−1.35, 0.10)	− 0.28
Proble solving skill against stress				
Baseline	3.47 (1.12)	3.14 (1.23)	–	–
Post-intervention	4.07 (0.70)	3.62 (0.87)	0.44 (−0.19, 1.07)	0.02
Self-reinforcement				
Baseline	2.47 (1.07)	3.14 (1.03)	–	–
Post-intervention	3.47 (1.13)	2.77 (0.73)	*1.01 (0.26, 1.76)*	0.58
Negative autonomic thought				
Baseline	3.24 (1.09)	2.50 (1.16)	–	–
Post-intervention	2.33 (1.18)	3.00 (1.15)	−0.61 (−1.58, 0.37)	−0.39

^a^Adjusted group differences were estimated using the method of Vickers & Altman [17]

^b^Standardized effect size (Cohen’s d) was calculated as the adjusted differences between intervention and control groups divided by the pooled within group standard deviation

A comparison of S-CBT and control group scores at baseline with those at the follow-up assessment revealed small to medium intervention effect sizes (range: d = 0.32–0.58) for “self-reinforcement” in both the high- and low-SWB groups after controlling for the effects of baseline scores. A medium effect size (d = 0.50) for “problem solving skills for alleviating stress” was observed only in the low-SWB group; among the high-SWB subjects, this change was not significantly greater in the S-CBT than in the control group (Table 2).

To examine the pre-to-post change in the scores of “problem solving skills for alleviating stress”, the general linear model (GLM) analysis was performed, including SWB (high = 1; low = 0) and interaction between SWB and intervention as independent variables for the entire sample. This showed that the pre-to-post score change was not significantly related to the “interaction between SWB and intervention” (parameter estimate, − 0.580; standard error, 0.473; *P* = 0.226), after adjusting for baseline scores on “problem solving skills for alleviating stress” (parameter estimate, 0.831; standard error, 0.116; *P* < 0.001), SWB (parameter estimate, 0.451; standard error, 0.734; *P* = 0.542), and intervention (parameter estimate, 0.965; standard error, 0.344; *P* = 0.007). When the GLM analysis was limited to those in the low-SWB group, the pre-to-post score change in “problem solving skills for alleviating stress” was significantly greater in the S-CBT group (parameter estimate, 0.753; standard error, 0.346; *P* = 0.043), even after adjusting for the baseline scores on “problem solving skills for alleviating stress” (parameter estimate, 0.852; standard error, 0.234; *P* = 0.002), the K6 (parameter estimate, 0.006; standard error, 0.042; *P* = 0.890), the Social Network Scale (parameter estimate, − 0.111; standard error, 0.056; *P* = 0.065), and age (parameter estimate, 0.085; standard error, 0.040; *P* = 0.049). However, no significant results for the pre-to-post score change in “problem solving skills for alleviating stress” were found in the high-SWB group.

Concerning K6 and the Social Network Scale, the pre-to-post score changes were not significant in either the high- or low-SWB group (Table 2).

## Discussion

In the present study, the 6-week S-CBT was effective in enhancing “self-reinforcement” cognitions among elderly men who were familiar with shogi, regardless of the participants’ SWB status. During the S-CBT sessions, each participant was instructed to face a variety of opponents with different shogi skill levels several times, and most participants experienced winning a game and receiving strong praise from the shogi instructor and others. According to behavioral science theory, the experience of being praised could induce a pump-priming effect to lift the person from a vicious cycle of failure and ‘excess disability’ to a virtuous cycle of willingness and the manifestation of potential capacity [21]. Being publicly praised and appreciated is a typical social reward. Social rewards also recruit the dopaminergic reward system and stimulate motivation [22, 23]. Thus, the nature of S-CBT is suited for instruction in self-esteem using practical situations.

In contrast, cognitions focused on “problem solving skills for alleviating stress” were significantly enhanced only in elderly men with low SBW; this finding remained significant after controlling for the effects of age and selected variables including baseline “problem solving skills for alleviating stress” scores, mental health, and social support. The reason for the discrepancy in results between the two SWB groups merits further discussion, although the interaction effects between SWB and intervention were not significant in the GLM procedure. During the S-CBT sessions, a variety of possible ideas intended to combat negative feelings were introduced using shogi-related situations such as “changing one’s mind when defeated”, which also has implications for stress management in daily life. In the present study, K6 scores were significantly greater in the low- than in the high-SWB group, suggesting that, at baseline, the low-SWB individuals were relatively likely to feel hopeless and to be unaware of ways to improve their mood [24]. Thus, it is possible that they might have become aware of appropriate ideas for stress management techniques that they could perform, whereas the high-SWB persons were already aware of such ideas before the S-CBT intervention. Low SWB is a potential risk factor for physical and psychological morbidities, including dementia and depression [25], and a future goal is to establish more effective S-CBT programs that are easily accepted by low-SWB persons.

The present study has several limitations. First, this was a non-blinded randomized controlled trial, so the data must be interpreted carefully. Although we obtained promising findings based on programs of collaboration between the psychologist and the shogi professional, the nature of the non-blinded study design requires that future studies consider variability in the characteristics of the trainers, such as the fluency of instruction, and of the trainees, such as the willingness to accept instruction. Second, with respect to the study controls, the study lacked an “attention” control, i.e., a generic psychosocial intervention providing non-specific attention, support, concern, and positive expectations. A Hawthorne effect may have occurred, wherein research staff may have made a greater effort to help those allocated to the control group after learning of the study. Specific shogi-assisted effects will be clarified by setting some psychological intervention as a control in future studies. The third limitation has to do with the generalizability of the findings. The subjects were all elderly men who were familiar with shogi, and they might already have had extant social networks, which could distinguish them from elderly people who engage in no recreational activities. It would be interesting to include shogi beginners in the S-CBT program and to clarify the feasibility of a program according to shogi levels and age categories, from children to elderly people, in studies with larger sample sizes.

Despite these limitations, our findings have several important implications for the application of CBT with elderly individuals. In the context of active aging, the goal of psycho-geriatric endeavors is to develop and evaluate interventions designed to stimulate improvement in subjective well-being, self-esteem, and friendship as well as to reduce loneliness among elderly individuals [11]. This S-CBT program is a promising learning tool that uses a board game as a form of leisure activity to modify negative and illness-prone thoughts and behaviors.

## Abbreviations

ANCOVA: Analysis of covariance
ANOVA: Analysis of variance
CBT: Cognitive–behavioral therapy
GLM: General linear model
S-CBT: Shogi-assisted CBT
SD: Standard deviation
SWB: Subjective well-being
