# Correction to: BPSM-D-19-00022R2 Subjective well-being and problem-solving skills for alleviating the stress of elderly men attending a randomized controlled trial of shogi-assisted cognitive behavioral therapy

**DOI:** 10.1186/s13030-019-0157-0

**Published:** 2019-06-25

**Authors:** Mutsuhiro Nakao, Hirokazu Furukawa, Chiho Kitashima, Shota Noda

**Affiliations:** 1 Department of Psychosomatic Medicine, School of Medicine, International University of Health and Welfare, 4-3, Kozunomo, Narita-shi, Japan; 2 School of Basic Research and Improvement of Practice for Education, Naruto University of Education, Tokushima, Japan; 3 Graduate School of System Design and Management, Keio University, Tokyo, Japan; 4 Graduate School of Human and Social Sciences, Musashino University, Tokyo, Japan

**Correction to: Biopsychosoc Med (2019) 13:11**

**https://doi.org/10.1186/s13030-019-0153-4**

In the original publication of this article [1], a system ID “BPSM-D-19-00022R2” is mistakenly included in the article’s title. The correct article tile should be “Subjective well-being and problem-solving skills for alleviating the stress of elderly men attending a randomized controlled trial of shogi-assisted cognitive behavioral therapy”.

The original article has been corrected.
